# The Association of Lipoic Acid Synthase (*LIAS*) Gene Methylation With Diabetic Kidney Disease

**DOI:** 10.1155/jdr/4342250

**Published:** 2025-11-28

**Authors:** Ziyi Feng, Ting Liu, Peng Gao, Yongwei Jiang, Meimei Zhao, Yi Liu, Haoyan Zhu, Mo Li, Nan Li, Xiaomu Kong, Liang Ma

**Affiliations:** ^1^China-Japan Friendship Institute of Clinical Medicine Research, China–Japan Friendship Hospital, Beijing, China; ^2^Department of Clinical Laboratory, China-Japan Friendship Hospital, Beijing, China

**Keywords:** diabetic kidney disease, DNA methylation, LIAS, oxidative stress

## Abstract

**Background:**

Diabetic kidney disease (DKD) is a leading cause of end-stage renal disease (ESRD). The dysregulation of lipoic acid synthase (*LIAS*) gene, which plays an essential role in the maintenance of mitochondrial function, was reported to participate in DKD pathogenesis. Therefore, the present study aimed to explore the association of *LIAS* gene methylation with the risk for DKD. DNA methylation has emerged as a potential biomarker for DKD.

**Methods:**

A cohort of 308 patients, comprising 156 patients with Type 2 diabetes mellitus (T2DM) with DKD (DKD group) and 152 with T2DM alone (T2DM group), were involved in the present study. A methylation-sensitive restriction endonuclease (MSRE)-qPCR approach combined with three enzymes (HpaII, AciI, and Hin6I) was developed to examine methylation patterns of the promoter region and exon 1 of *LIAS* gene. Logistic regression analysis was performed to evaluate the associations between methylation levels and DKD risk, and then the clinical conventional confounders, including age, sex, BMI, hypertension, and lipid profiles, were adjusted.

**Results:**

We successfully identified the methylation sites that can be recognized and digested by HpaII, AciI, and Hin6I, in the CpG islands of *LIAS* promoter region and exon 1. Applying the MSRE-qPCR approach, significant differences in methylation levels were observed at two CpG sites. Compared with T2DM group, the P3 site in the promoter region exhibited increased methylation level in the DKD group (5.886% vs. 10.229%, *p* = 0.043), whereas E1 site in exon 1 showed reduced methylation level in the DKD group (11.785% vs. 6.250%, *p* = 0.023). Furthermore, logistic regression revealed that increased methylation at P3 was associated with increased DKD risk [OR (95% CI) 1.029 (1.011, 1.047), *p* = 0.002], whereas increased methylation at E1 demonstrated a protective effect [OR (95% CI) 0.940 (0.896, 0.987), *p* = 0.013]. Then, via including P3 and E1 sites in the same model, we found both sites independently influenced the risk for DKD. In addition, these associations were not significantly altered after adjusted for the confounders.

**Conclusion:**

The findings indicate that *LIAS* gene methylation participate in DKD pathogenesis, providing novel insights into its pathophysiological mechanisms. It highlighted that *LIAS* methylation could serve as a promising biomarker for DKD. Also, it supported the utility of MSRE-qPCR in clinical applications.

## 1. Introduction

Diabetic kidney disease (DKD) is a serious microvascular complication of diabetes, affecting up to 40% of patients, as reported by the International Diabetes Federation [1]. Global epidemiological data estimated 6.3 million cases of DKD attributable to Type 1 diabetes and 107.6 million attributable to Type 2 diabetes mellitus (T2DM) in 2021. By 2030, these numbers are projected to increase to 7.4 million and 129.8 million, respectively [2]. Including a large number of patients who will require dialysis and/or kidney transplantation about one-third of patients may eventually progress to end-stage renal disease (ESRD), bringing a huge economic burden to the world [2]. Studies have shown that the burden of chronic kidney disease related to diabetes in China is continuing to increase, measured by mortality and disability-adjusted life years (DALYs) [3]. The diagnosis of DKD is based on renal pathological biopsy, which is the gold standard. Still, because it is an invasive examination and potentially harms patients, its clinical application is limited. In clinical practice, estimated glomerular filtration rate (eGFR) and urinary albumin excretion are often used as predictors of kidney disease progression, cardiovascular disease (CVD), and mortality in patients with DKD [4]. However, their prognostic significance is not specific to DKD. Therefore, it is urgent to explore novel and specific biomarkers of DKD, to deepen the understanding its pathogenesis and to promote its early diagnosis and treatment.

DNA methylation is the earliest discovered and most widely studied epigenetic mechanism, which can regulate the expression level of genes without changing DNA sequence [5]. Previous studies have shown that DNA methylation is involved in the pathogenesis of DKD and has the potential to serve as a new diagnostic and therapeutic biomarker for DKD [6]. For example, a study found that there are significant differences in DNA methylation patterns between patients with diabetes and patients with DKD [7]. Based on the analysis of methylome data, we previously found that the methylation level of the promoter region of the lipoic acid synthase (*LIAS*) gene, a key enzyme responsible for lipoic acid (LA) synthesis, was significantly upregulated in the renal tissue of the DKD rat model, suggesting that it may be involved in the pathophysiological mechanism of DKD. *LIAS* is a key enzyme involved in the synthesis of LA, which plays an important role in the key energy metabolism process in mitochondria [8]. It plays an important role in the LA cycle of tricarboxylic acid cycle, and is closely related to the antioxidant mechanism [9]. Previous studies also suggested that methylation levels of *LIAS* can influence oxidative stress by regulating gene expression and participating in the occurrence and development of various diseases. For example, the methylation level of *LIAS* promoter region in renal clear cell carcinoma tissue was increased, whereas the methylation level of *LIAS* in gastric carcinoma tissue was decreased [10, 11].

In the present study, we investigated the association of *LIAS* gene methylation with the risk for DKD for the first time in a Chinese population. Via developing a methylation-sensitive restriction endonucleases (MSRE)-qPCR method, we examined the methylation levels of CpG islands in *LIAS* gene promoter and exon 1 in patients with diabetes with and without DKD. It revealed that patients with DKD showed significant alterations of methylation levels in *LIAS* gene when compared with those patients without DKD. It highlighted the potential role of *LIAS* gene methylation in the pathogenesis of DKD, and it could be a promising biomarker for DKD in the future.

## 2. Method

### 2.1. Participants

We conducted a case–control study with patients diagnosed with T2DM at the China-Japan Friendship Hospital from October 2022 to December 2023. A total of 152 patients with T2DM without DKD (as T2DM group) and 156 patients with T2DM with DKD (as DKD group) were enrolled in this study. Diabetes was identified according to the ADA classification and diagnosis of diabetes (2023 Edition) [12], and DKD was identified by the Chinese guidelines for the clinical diagnosis and treatment of DKD [13].

The inclusion criteria for T2DM group is as follows: (1) aged 40 to 80 years; (2) diagnosed with T2DM; (3) urine albumin–creatinine ratio (UACR) < 30 mg/g and eGFR > 90 mL·min^−1^·(1.73 m^2^)^−1^. For the DKD group, the inclusion criteria are the following: (1) aged 40 to 80 years; (2) diagnosed with T2DM; (3) evidence of DKD was required, defined by persistent albuminuria (UACR ≥ 30 mg/g or 24-h urinary albumin excretion > 0.3 g) confirmed on two occasions over 6 months, or a reduced eGFR (< 60 mL·min^−1^·(1.73 m^2^)^−1^), or renal biopsy findings indicative of DKD.

Patients will be excluded from the study if they meet any of the following criteria: (1) History of major macrovascular events in the past 6 months; (2) suffering from severe complications related to Type 2 diabetes; (3) suffering from other primary or secondary renal diseases, blood diseases, malignancies, or severe systemic diseases; (4) experiencing active infections in the past 4 weeks; (5) having severe psychological or mental health problems; or (6) pregnant or lactating women.

### 2.2. Ethical Statement

This study was in accordance with the Declaration of Helsinki II and approved by the clinical research ethics committee of the China-Japan Friendship Hospital (Identifier: 2024-KY-0325). The research adhered to local laws and institutional requirements, with written informed consent obtained from all participants.

### 2.3. Data Collection

Participants were asked to complete a structured questionnaire to collect demographic information, including age, sex, body weight, height, smoking, and drinking history. Body mass index (BMI) was calculated by dividing weight (kg) by height squared (m^2^). Biochemical parameters were analyzed: urea, creatinine (Cr), uric acid, glucose, total cholesterol, triglycerides, high-density lipoprotein-cholesterol (HDL-C), and low-density lipoprotein-cholesterol (LDL-C) using assay kits on the AU5800 Chemistry Analyzer (Beckman Coulter, Brea, CA, United States). Urinary albumin excretion was measured by immunoturbidimetry on the UniCel DxC800 (Beckman Coulter, CA, United States). Then, urinary albumin/creatinine ratio (UACR) was calculated. Hemoglobin A1c (HbA1c) was evaluated using the Mindray glycosylated hemoglobin meter (Mindray, Shenzhen, China).

### 2.4. Extraction of DNA

DNA was extracted from peripheral blood using Tianlong GeneRotex 96 (Xi'an Tianlong Science and Technology Co. Ltd., Xi'an, China). We used NanoDrop 2000 (Thermo Fisher Scientific, United States) to assess the quality of the extracted DNA to ensure that the OD_260_/OD_280_ ratio was between 1.8 and 2.0. The extracted DNA samples were stored at −70°C before use.

### 2.5. DNA Methylation Detection

We developed a methylation-sensitive restriction endonucleases (MSREs)-qPCR method to detect methylation of *LIAS* gene. In brief, the DNA digestion was performed using MSREs obtained from New England Biolabs (MA, United States) and Thermo Fisher Scientific (MA, United States). Three enzymes with distinct recognition sequences were selected to increase the coverage of CpG sites: AciI (5′-CCGC-3′, R0551S, New England Biolabs, MA, United States), Hin6I (5′-GCGC-3′, FD0484, Thermo Fisher Scientific, MA, United States), and HpaII (5′-CCGG-3′, ER0512, Thermo Fisher Scientific, MA, United States). These enzymes specifically recognize CpG motifs within their respective recognition sites and selectively digest unmethylated DNA, leaving methylated DNA unaffected. The use of multiple MSREs ensured broader detection of methylation-sensitive regions.

For the digestion experiments, to ensure optimal enzyme activity, each sample's DNA was separately digested with each enzyme in their respective specific buffers. Each digestion reaction contained 200 ng of input genomic DNA and 4 U of one of the three enzymes in a total reaction volume of 20 *μ*L, prepared in their corresponding matching buffers. The reaction volume was adjusted to 20 *μ*L with nuclease-free water. The digestion mixtures were incubated at 37°C for 5–15 min in a standard thermal cycler (Bio-Rad Laboratories, Inc., CA, United States) following the manufacturer's instruction. After incubation, the reactions were heated to 65°C for 20 min to inactivate enzymes and then cooled to 4°C to preserve the DNA for subsequent analyses. Meanwhile, undigested control experiments were performed following the same procedure, in which enzyme was replaced by DNA-free water. Then, the processed DNA templates were subsequently used for MSRE-qPCR to quantify methylation-sensitive loci.

Quantitative PCR (qPCR) was performed to analyze the digested and undigested control DNA templates using a SLAN-96P fluorescent quantitative PCR system (Hongshitech, Shanghai, China). Prior to amplification, the enzyme-digested DNA was diluted to a final volume of 200 *μ*L. The primers of each specific target region were synthesized by Beijing Tsingke Biotech Co. Ltd. (Beijing, China), as detailed in Table 1. Two primer pairs were designed to amplify the promoter region of *LIAS* gene, while the other two pairs targeted the exon 1. Each primer pair was amplified in a separate PCR reaction. Each PCR reaction contained 5 *μ*L of the diluted DNA, 12.5 *μ*L of Ultra SYBR Mixture (CW0957, Jiangsu Cowin Biotech Co., Ltd, Jiangsu, China), 0.2 *μ*M forward primer and 0.2 *μ*M reverse primer in a 25 *μ*L total volume.

After optimization, the qPCR protocol included an initial denaturation step at 95°C for 10 min, followed by 45 cycles of denaturation at 95°C for 15 s, annealing at 60°C for 30 s with fluorescence acquisition, and extension at 72°C for 30 s. The methylation level at each site was quantified based on the real-time fluorescent signal. Post-amplification, melting curve analysis was conducted to confirm the specificity of the PCR products. This involved holding the reactions at 95°C for 15 s, 60°C for 1 min, and then gradually increasing the temperature from 60°C to 95°C at a rate of 0.08°C/s, with a final hold at 95°C for 15 se.

The percentage of 5-mC modification (Met) of each site was calculated using *Δ*Ct method based on the difference in Ct values derived from the fluorescent signal in qPCR between digested template and undigested control template of the same individual, using the formula:

 $$\begin{array}{c} \text{Met}=2-\left(\text{Ct digested}-\text{Ct undigested}\right)\times 100\% \end{array}$$

### 2.6. Data Management

The sample size was determined a priori using G∗Power to achieve a statistical power of 80% at a significance level of 0.05, assuming an effect size of 0.56 based on previous studies or pilot data. An equal allocation ratio (1:1) was used for the two groups. To address missing data, we implemented complete-case analysis. In this study, we took several measures to minimize potential sources of bias. Random sampling was employed to address selection bias. To address potential confounders, key variables were adjusted for in the regression models. Additionally, sensitivity analyses were conducted to evaluate the robustness of the findings.

### 2.7. Data Analysis

Data are shown as *n*(%), mean ± standard error (SD) or median (interquartile range). Chi square test was used for the comparisons of categorical variables, while independent sample *t*-test was used to compare the continuous variables with normal distribution. For those data that does not follow a normal distribution, nonparametric Mann–Whitney *U* test was used. Binary logistic regression was used to test the association between methylation ratios with the risk for DKD. The odds ratio (OR) and 95% confidence interval (CI) were described as per 1% increment of DNA methylation ratio. All data were analyzed using SPSS 22.0 software (IBM Corporation, NY, United States). *p* < 0.05 was considered statistically significant.

## 3. Result

### 3.1. Clinical Characteristics

The clinical information indicates no significant differences in gender distribution, age, BMI, blood glucose levels, or routine lipid parameters between the T2DM and DKD groups. As expected, renal function markers such as serum urea, serum creatinine, eGFR, urinary albumin excretion, and UACR showed significant differences, with the DKD group exhibiting worse renal function (Table 2).

### 3.2. Methylation Regions and Sites Selection

**Figure 1:**
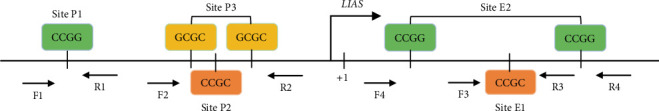
Schematic diagram of methylation sites in *LIAS* promoter region and exon 1.

Using UCSC network online tools (http://genome.ucsc.edu), we analyzed and determined the CpG islands in *LIAS* promoter region and exon 1 region. As shown in Figure 1, P1 and E2 methylation sites (green blocks) can be recognized and cleaved by HpaII. P2 and E1 methylation sites (red blocks) can be targeted by AciI. P3 methylation site (orange blocks) can be targeted by Hin6I.

### 3.3. Methylation Ratios Analysis

As shown in Table 3, the methylation ratio of P3 site was significantly higher in DKD group compared with T2DM group, whereas the methylation ratio of E1 site in DKD group was significantly lower than that in T2DM group. In addition, the methylation ratios of P1, P2, and E2 in T2DM group and DKD group were comparable (Table 3).

### 3.4. Association Between Methylation Ratios of P3 and E1 Sites With Risk for DKD

As shown in Table 4, logistic regression analysis showed that per 1% increment of methylation ratio of P3 site was positively associated with increased risk for DKD with an OR of 1.029 (*p* = 0.002), while per 1% increment of methylation ratio of E1 was negatively associated with the increased risk of DKD with an OR of 0.940 (*p* = 0.013). Further, we adjusted the conventional clinical confounders for DKD including age, sex, BMI, hypertension, total cholesterol, triglycerides, HDL-C, and LDL-C in the logistic models. The results suggested the associations between these sites and DKD remained significant, and not significantly altered (Model 2). Then, we included both P3 and E1 in the model, and the results showed that both factors remained significant. This indicates that the methylation levels of P3 and E1 are independent risk factors for DKD.

## 4. Discussion

DKD is one of the major causes of ESRD and an important cause of morbidity and mortality in patients with diabetes [14]. The pathogenesis of DKD is extremely complex, involving the interaction of multiple factors such as metabolic disorders, hemodynamic abnormalities, and inflammatory pathways [15]. Chronic hyperglycemia can induce oxidative stress, accumulation of advanced glycation end products (AGEs), and activate pro-inflammatory and fibrotic signaling pathways, leading to glomerular and tubular damage [16]. However, despite the continuous deepening of related research, the specific molecular mechanisms driving the progression of DKD are still not fully understood, which limits the development of new markers and targeted therapies. At present, the widely used markers in clinical practice include urinary albumin excretion rate and eGFR, but these indicators are insufficient in specificity and sensitivity, especially in early disease detection or prediction of disease progression [17]. This gap in clinical needs has prompted researchers to seek new molecular markers that can reflect the mechanism of the disease. Among them, epigenetic modifications such as DNA methylation have received increasing attention due to their role in regulating gene expression and potential reversibility, and have become a hot spot for marker development and therapeutic target research [18].

This study focused on the promoter and exon regions of the *LIAS* gene to analyze its DNA methylation pattern and its relationship with the occurrence and development of DKD. Given the key role of *LIAS* in LA synthesis and redox balance, our study revealed a potential link between its epigenetic abnormalities and the pathological mechanism of DKD. In addition, we found that changes in specific methylation sites in the promoter and exon-1 region of *LIAS* gene have the potential to become potential biomarkers. These methylation signatures can be used to capture epigenetic profiles associated with DKD, thereby serving as early diagnostic markers or progression predictors of the disease.

Epigenetic modifications, particularly DNA methylation, have emerged as promising biomarkers for DKD because of their unique advantages over traditional clinical indicators [19]. Unlike conventional biomarkers such as albuminuria and eGFR, which reflect renal damage only at advanced stages, DNA methylation patterns can capture early molecular alterations linked to disease pathogenesis [20]. Moreover, DNA methylation is highly stable in various biological specimens (e.g., blood and urine) and can be detected using sensitive, high-throughput technologies such as bisulfite sequencing and methylation-specific PCR, facilitating its potential clinical application [21]. Several studies have emphasized the association between DNA methylation changes and the progression of DKD. For example, differential methylation of *PQLC2*, *PTPN6/PHB2*, and *PRPF8* has been reported to be associated with fibrosis and inflammation leading to kidney damage [22]. In addition, changes in methylation levels of *Sod1/2*, *OGG1*, and *GSTP1* are associated with oxidative stress and mitochondrial dysfunction related to DKD [23].These findings underscore the relevance of epigenetic regulation in DKD and its potential utility in identifying novel molecular markers. Despite considerable advancements, the clinical translation of DNA methylation biomarkers faces significant limitations. Key challenges include small sample sizes, subtle effect sizes, cellular heterogeneity, and confounding factors, which together hinder the identification of consistent and reproducible methylation sites across diverse populations and sample types [24]. Additionally, phenotypic heterogeneity and a lack of replication studies further complicate efforts to link differentially methylated regions (DMRs) to specific traits. The functional relevance of many DMRs remains poorly understood, and their causal relationship with the progression of DKD is yet to be clarified [25]. Therefore, identifying methylation sites that are not only disease-associated but also predictive of DKD onset or response to therapy, as well as developing the corresponding methylation detection technologies, are crucial for improving DKD diagnosis, risk stratification, and therapeutic intervention.

*LIAS* plays a pivotal role in mitochondrial function by encoding LA synthase, an enzyme essential for synthesizing LA a powerful antioxidant. LA facilitates the LA cycle, scavenging reactive oxygen species (ROS), and regenerating other antioxidants such as glutathione [26]. This process is crucial for maintaining mitochondrial homeostasis, especially under oxidative stress. Mitochondria are central to kidney function, providing the energy required for glomerular filtration, tubular reabsorption, and secretion [27]. Consequently, mitochondrial dysfunction is increasingly recognized as a key mechanism in the development of DKD. Impaired mitochondrial function leads to excessive ROS production, disrupted energy metabolism, and the activation of inflammatory and fibrotic pathways, which collectively contribute to renal damage [28]. Dysregulation of *LIAS*, through either genetic or epigenetic mechanisms, exacerbates mitochondrial dysfunction, heightening oxidative stress, and promoting the pathogenesis of DKD [29]. Previous studies demonstrated that alterations in *LIAS* expression impair mitochondrial dynamics, hinder the electron transport chain, and amplify oxidative damage, thereby exacerbating renal injury [30].

DNA methylation, as a key epigenetic modification, has emerged as an important regulatory mechanism affecting *LIAS* expression. Aberrant methylation of *LIAS* suppresses its transcription, reducing LA synthesis and undermining mitochondrial antioxidative capacity [31]. In the present study, we initially utilized the bioinformatic tools to predict the CpG islands in *LIAS* gene. In common with the majority of genes, dense CpG islands are only predominantly clustered in the promoter and exon 1 regions. This finding indicates that these regions may serve as critical candidate loci for methylation-based regulatory. Moreover, according to the current understanding, methylation in the promoter region generally leads to gene silencing, whereas methylation in the gene body (such as exons) functions in maintaining transcriptional elongation, mRNA stability, and regulating alternative splicing, suggesting distinct gene regulatory mechanisms between promoter and exon methylation. Given these reasons, we selected both the promoter and exon 1 regions for further methylation analysis.

Regarding the results of this clinical study, in the DMRs selected for the promoter and exon regions of the *LIAS* gene, the results of the MSRE-qPCR experiment showed that there were significant differences in the methylation levels of the P3 and E1 regions between the T2DM group and the DKD group. To further analyze the impact of changes in the methylation ratio on the occurrence of DKD, we performed a logistic regression analysis. It is shown that both methylation sites contributed independently to the risk for DKD, even after adjusted for the conventional clinical confounders including age, gender, BMI, and blood lipids. In Model 1, only P3 or E1 methylation ratio was used as an independent variable. The results showed that a 1% increase in P3 methylation ratio increased the risk of DKD by 2.9%, while an increase in E1 methylation ratio reduced the risk of DKD by 6.0%. To further adjust the model, age, gender, BMI and lipid-related indicators (CHO, TG, HDL-C, LDL-C) were added for logistic regression analysis. The adjusted Model 2 showed that the P3 and E1 methylation ratios still had a significant impact on the occurrence of DKD, and the results were similar to those in Model 1.

Furthermore, the results indicated increased methylation of P3 as a risk factor for DKD, and increased methylation of E1 as a protective factor. DNA methylation in different parts may have different effects on gene expression regulation. Methylation in the promoter region may inhibit gene expression, while methylation in the exon region may cause gene expression to increase. Thus, we speculated that increased methylation of P3 in the *LIAS* promoter region reduced LA content by inhibiting *LIAS* expression and weakening the ability to resist oxidative stress, thereby promoting the occurrence of DKD; whereas increased methylation of E1 in the *LIAS* exon region may increase LA content by upregulating *LIAS* expression and resisting oxidative stress, thereby playing a protective role in the occurrence of DKD. This inference is also consistent with previous research findings that LIAS overexpression significantly increased the antioxidant capacity in animal models, while LIAS inhibition led to reduced LA content, enhanced oxidative stress, and exacerbated DKD symptoms in these models [32]. After all, it is suggested a potential mechanism of *LIAS* gene methylation in DKD pathogenesis.

This study has following strength. Firstly, it explored for the first time the relationship between *LIAS* gene methylation and the risk of diabetic nephropathy (DKD), providing a fundamental understanding of the new epigenetic mechanism of this gene in DKD. Secondly, by targeting both the promoter and exon regions of the *LIAS* gene, this study obtained methylation information from functionally important gene regions, which provide a new perspective for understanding the regulation of the *LIAS* gene and its potential connection with the pathological mechanism of DKD. Finally, the methylation-sensitive restriction endonuclease (MSRE)-qPCR method used in this study is a stable, convenient, and accurate methylation detection method. Compared with the complex bisulfite conversion and sequencing methods, this technology has the advantages of low cost and simple operation, and can efficiently detect specific methylation sites. By combining three different restriction endonucleases, this study covered the methylation sites in the target region to the greatest extent, improving the accuracy and depth of the test results. The results provide an important basis for the development of *LIAS* gene methylation as a new DKD marker, and also lay the foundation for the further development of epigenetic-based diagnostic tools.

There are also some limitations. First, the sample population is from a single center in China. To draw more universal conclusions, future studies should expand the sample size and include data from multicenter and multiethnic groups. Second, since this study is a case–control study, it cannot explore any causal relationship of the epigenetic changes and DKD. In the future, functional experiments and mechanistic studies are warranted to further clarify the role of *LIAS* methylation in DKD pathology. Finally, as a method based on MSRE-qPCR, this method is limited by the specificity of the enzyme used; thus, some sites are not able to be detect in the present study. However, this study significantly alleviated this limitation through the design of a multienzyme combination to cover as much as possible methylation sites, indicating the robustness and applicability of this method in targeted methylation analysis. In addition, future studies that can overcome the current limitations will further enhance the clinical application potential of this method and expand its applicability in different populations and diseases.

## 5. Conclusion

This study establishes that methylation of the *LIAS* gene is associated with the risk of DKD, emphasizing its potential as a novel epigenetic biomarker. By using a robust detection method covering promoter and exon regions with three enzymes, it provides a reliable approach for methylation analysis. These findings highlight a foundation for future clinical applications and a better understanding of DKD pathogenesis.

## Figures and Tables

**Table 1:** Sequences of primers.

**Amplicon**	**Forward primer**	**Reverse primer**	**Product length**
1	F1: CGAACTCCCACTCAGCCCAA	R1: ACCTTGTCCTGTTTGTCCCC	161 bp
2	F2: CCCACAACAGGATTCGCATC	R2: GATGTTTCTGTGCTCGTGGG	158 bp
3	F3: CTGCACTGCGCTAGTCCTAA	R3: TATTATTGGGCATGGGCGCT	148 bp
4	F4: GCACGTAAGGCTAAAGCAGC	R4: GCGCTCTGAAGGGATAGGAT	241 bp

**Table 2:** Clinical characteristics of the study population.

**Variables**	**T2DM group**	**DKD group**	**p**
*N*	152	156	
*N_male_* (%)	105 (69.20)	100 (64.20)	0.581
Age	58.35 ± 9.88	62.45 ± 11.23	0.490
BMI	25.36 ± 3.54	25.70 ± 5.33	0.146
Fasting plasma glucose, mmol/L	8.50 ± 3.89	8.28 ± 2.86	0.411
Hemoglobin A1c, %	7.60 ± 1.69	7.77 ± 1.92	0.490
Total cholesterol, mmol/L	4.37 ± 1.16	3.97 ± 1.34	0.110
Triglycerides, mmol/L	1.85 ± 1.33	1.76 ± 0.87	0.603
HDL-C, mmol/L	1.25 ± 0.32	1.17 ± 0.35	0.244
LDL-C, mmol/L	2.71 ± 0.85	2.45 ± 0.96	0.146
Serum urea, mmol/L	5.68 ± 1.32	12.24 ± 6.03	**~0.001**
Serum creatinine, *μ*mol/L	64.75 ± 13.16	236.13 ± 242.23	**~0.001**
Serum uric acid, *μ*mol/L	349.33 ± 88.17	366.04 ± 99.12	0.364
eGFR, mL/min/1.73 m^2^	99.30 ± 10.61	44.95 ± 31.95	**~0.001**
Urinary albumin excretion, mg/L	4.05 ± 9.71	583.76 ± 925.06	**~0.001**
UACR, mg/g	2.65 ± 6.01	1008.81 ± 2032.67	**~0.001**

*Note:* Data are shown as *n* (%) or mean ± SD. Results with *p* < 0.05 are denoted in bold.

Abbreviations: eGFR: estimated glomerular filtration rate; HDL-C: high-density lipoprotein-cholesterol; LDL-C: low-density lipoprotein-cholesterol; UACR: urinary albumin/creatinine ratio.

**Table 3:** Comparisons of methylation ratios between T2DM and DKD groups.

**Site**	**T2DM group (%)**	**DKD group (%)**	**p**
P1	7.484 (3.504, 15.431)	8.901 (4.844, 11.785)	0.536
P2	20.637 (10.404, 30.320)	18.063 (9.572, 27.756)	0.378
P3	5.886 (2.947, 14.240)	10.229 (4.084, 93.790)	**0.043**
E1	11.785 (5.489, 18.686)	6.250 (2.693, 16.790)	**0.023**
E2	10.626 (1.635, 47.886)	12.243 (1.827, 38.852)	0.925

*Note:* Data are shown as median (interquartile range). *p* < 0.05 is denoted in bold.

**Table 4:** Logistic regression analysis of methylation ratios and DKD.

**Independent variable**	**Model 1**		**Model 2**	
	**OR (95% CI)**	**p**	**OR (95% CI)**	**p**
P3	1.029 (1.011, 1.047)	**0.002**	1.037 (1.010, 1.065)	**0.007**
E1	0.940 (0.896, 0.987)	**0.013**	0.931 (0.881, 0.984)	**0.012**
(Include both P3 and E1 as independent variables)				
P3	1.034 (1.014, 1.055)	**0.001**	1.042 (1.016, 1.069)	**0.001**
E1	0.917 (0.864, 0.964)	**0.005**	0.913 (0.855, 0.975)	**0.006**

*Note:* The OR and 95% CI are described as per 1% increment of DNA methylation ratio. Model 1: unadjusted; Model 2: adjusted for age, sex, BMI, history of hypertension, total cholesterol, triglycerides, HDL-C, and LDL-C. *p* < 0.05 is denoted in bold.

## Data Availability

All data generated or analyzed during this study are included in this article.
